# The Nature of Nursing Sore Mouth

**Published:** 1855-07

**Authors:** M. L. Knapp


**AN INQUIRY INTO THE NATURE OF THE ANOMALOUS AFFECTION KNOWN BY THE APPELLATION OF "NURSING SORE MOUTH," OR "PUERPERAL ANÆMIA." By M. L. Knapp, M. D.**

I. Present State of Medical Knowledge.—Views of Correspondents.—Abstract of Literature.—The Writer's Views of the Nature of the Affection.

THE term Nursing Sore Mouth is so well understood to mean a peculiar form of disease to which suckling women are subject, that its adoption, though it be popular rather than professional, cannot lead to any mistake. There may be some practitioners who have never encountered this disease at the bed-side, and possibly those who have never heard of it; yet there are numerous physicians who have met with it and found it a very serious and obstinate affection. The silence in the main of standard or systematic authors on the subject; the omission in the arrangements of the best nosologists of any affection of the mouth peculiar to lying-in women characterized by the phenomena that are manifested in this complaint; and the testimony of the Journals, nevertheless, that such an affectien does exist, is often met with, and is greatly on the increase in some localities; render it, most decidedly, a proper subject of inquiry, particularly in the absence of any clear or satisfactory views from those under whose observation it has more frequently fallen, as to its nature, pathology, and rational method of cure. To deny that a peculiar, lingering form of disease, often of very grave severity, characterized by anæmia, debility, and other phenomena of which soreness of the mouth constitutes a prominent local symptom, now and then attacks women in the state of lactation, and persists, sometimes, in spite of all the remedial measures brought to bear upon the case, finally either proving fatal or terminating in a slow and gradual recovery incident to circumstances, as the removal of the infant from the breast, change of season, etc., rather than the administration of medicines, would be to shut our eyes to facts and experience. The historyof hundreds of such cases is annually unwritten, willingly oblivionized among the unrecorded transactions of groping practice: and yet these cases are not wholly lost, for they reach us from the nursery as the reminiscenses of many a delicate mother's past sufferings, and forebodings of their re-occurrence, constituting some of the oral literature of this dreaded affection. There are few practitioners, we opine, who have altogether escaped these popular traditions of the Nursing Sore Mouth affection.

The recorded literature of this disease is made up of the brief articles on this subject that have from time to time appeared in the medical Journals. Some of these are mere notices that such an affection has been met with, accompanied with the recommendation of a favorite remedy, perhaps, while others contain well-marked and well-drawn-up cases of the disease. We propose as a starting point in our inquiry to canvass what is known and what has been published on the subject, so far as we may be able to reach the files of the Journals. It is probable an article may here and there be overlooked, but sufficient testimony will appear to establish very clearly the grave character of the malady; that it is nowhere understood, every where treated empirically; and that it has hitherto failed to receive such careful investigation at the hands of any member of the profession as to settle the question of its pathology and treatment. Prior to glancing into the Journals, however, we will offer the original views of our correspondents, elicited by the following circular, two hundred and fifty copies of which were distributed to leading practitioners of the United States. We will not marvel that only three medical gentlemen have responded.— These three contributions on the subject, however, are valuable.— They establish the grave character of the malady, the want of any exact knowledge of its true character, and the further interesting facts that it has puzzled, perplexed and engaged the attention of the profession in sundry parts, and that it is looked upon as a subject of sufficient general interest to demand a searching inquiry. Our acknowledgments are hereby tendered to the courteous gentlemen who kindly responded to the call.

*Circular.—Cincinnati, Dec.* 22, 1853.—DEAR SIR: My apology for trespassing on your time and attention is the following, to wit: I am instituting some inquiries into the nature and history of that anomalous affection known in the United States by the popular designation of "*Nursing Sore Mouth.*" Attention has been briefly called to this disease through some of the Journals as a form of "*puerperal anæmia.*"

Having encountered the disease in localities widely apart, and having conversed with physicians in different States who had met with it frequently; I am led to think that it occurs more or less in all the States and British Provinces, but more frequently, by far in some localities than others. Although it is believed to occur thus extensively, and so frequently during some years that it may be said to be a not uncommon form of disease, still little is understood of it except empirically, and it is consequently treated with very variable results, the majority of well-marked cases proving obstinate, running a course of some months, and in many instances yielding only with a snail's pace, after the removal of the puny infant from the breast, which dernier resort seems to be a *sine qua non* in the recovery of inveterate cases.

Its literature, I think, is limited to a few fragmentary notices, that have appeared in the Journals within the last few years: nothing is said of this obstinate and sometimes fatal form of disease in our elaborate treatises on Practice and Diseases of Women.

Presuming that this anomalous and nondescript form of disease has come under your notice, and that you will feel an interest in contributing to its elucidation, I respectfully request you to call to mind your cases of "nursing sore mouth," and the impressions they have left on your mind, and, at your earliest convenience, to draw up and forward to me by mail your observation and experience on the subject. My object is to arrive at just conclusions by means of more extended data than my own observations afford. This appears to me to be a legitimate mode of investigating the subject, and the only practicable method perhaps, for the disease seems to shun the lying-in wards of hospitals (or we should have heard something of it,) and to occur wholly in private practice.

Should I be so fortunate as to arrive at any practical deductions deemed of general interest to the profession, and worthy of publication, the condensed views or points of practical importance (and, if not too copious, the views in full) of my correspondents will also appear, that each may receive the award of merit due to his own observations. Allow me to add in this connection that it will give me pleasure to reciprocate the favor at any time you may call upon me for a like civility.

Having made known my object and wishes in a general way, I now take leave to call your attention to a few points in detail I wish your answer to cover, which, for your more convenient reference, I have numbered.

1. Topography of your region of country, and how long settled; agriculture and products; state of horticulture and orcharding; general character of disease?

2. Number of years in practice; number of cases of nursing sore mouth met with; history, symptoms, treatment, duration, result, of one or two cases as types?

3. If death has resulted in any case, the mode or manner in which it took place?

4. The effect of the disease in the mother on the child; if the infant died, or was removed from the breast, the effect of a suppression of lactation on the progress of the case?

5. Whether cases have occurred more frequently in some years than others; if so, the character or constitution of those years as to temperature, snow, rains frosts; whether the crops were blighted or short; season of its development?

6. Whether attacks have occurred oftener after first confinement than in subsequent ones?

7. Whether attacks occur oftener after very severe labors, flooding, or other prostrating accidents of delivery?

8. Whether you have known an attack follow abortion, or parturition where the infant was still-born, or was removed from the breast and lactation suppressed?

9. Whether the disease has ever to your knowledge made its incursion before delivery; and if so, the effect of parturition on the case?

10. Whether, within the range of your observation, other females than those in a pregnant, puerperal, or suckling state, have suffered an attack of this form of anæmia; and if so, whether concurrently in the same family where a nursing female was laboring under it?

11. Whether, according to your observation, it be a disease peculiar to women; or have you met with the same morbid diathesis and assemblage of symptoms in males, during years of the greater prevalence of this disease?

12. Whether you have noticed in seasons of the more frequent occurrence of this affection, a prevalent morbid diathesis that seemed to aggravate and render more intractable the common forms of disease?

This covers all the points I wish to have categorically responded to; but any views of your own—any facts or deductions from your practical experience and observation, throwing light on the etiology, pathology, or treatment of this affection, will be thankfully received.

*Dr. Ellsworth's Reply.—Hartford, Jan.* 21, 1854.—DEAR SIR: Many duties have hitherto prevented an answer to your favor of the 22d December. Your letter was handed to a physician having more extensive practice than myself in the department of midwifery, but as he insisted on my writing, you shall have all the information I possess: the questions shall be answered briefly, at least as many of them as it is in my power to answer.

1. Hartford is partly built on the alluvial of the Connecticut River Valley, but the country is mainly primitive. The farms are rich, highly cultivated, and possess good depth of soil. Diseases in our valley generally assume a typhoid type, and do not bear the lancet as well as in higher portions of the State, and my impression is that V. S. is not as well borne as some years since.

2. My present experience extends back only fifteen years. The disease under consideration is not of very frequent occurrence, though common enough to excite earnest desire for its amelioration. The treatment, symptoms, etc., have been discussed by our City Medical Society.

3. As a general thing patients have recovered, though weaning has occasionally been necessary to effect this. I have known no case of death from the disease alone.

9. I had a very severe case commencing nine weeks prior to labor; the patient had nearly died from the disease with a previous child, in which case also the complaint made its appearance prior to delivery. She recovered, and I think without removing the child.

The treatment is simple, consisting of good support by way of food, bark with lime-water, carb. ferri, carb. sodæ, and particularly porter. Almost everything tonic is useful, but especially the articles mentioned.

The minutes of our Society present but little worth mentioning in addition, except a statement made by Dr. Miner that he knew a severe epidemic of it in Berkshire Co., a mountainous region in Massachusetts, in 1832, and another at Middletown, in Connecticut, in 1836. Dr. Sumner also stated that he found persons subject to this complaint more disposed to phthisis. He had known the disease to occur as early as the fifth month of pregnancy. Local treatment does not appear to be particularly serviceable. Some of the questions remain unanswered, because I must either give a negative answer or one of no particular service to you. Hoping what is recorded may be useful to you, I remain, yours, truly,

P. W. ELLSWORTH.

*Remarks.*—Dr. Ellsworth's contribution establishes the fact very clearly that the disease in question is not confined to the period of lactation, for he observed its occurrence twice in the same female, and on both occasions it made its incursion before delivery. Dr. Sumner also had observed it as early as the fifth month of pregnancy. It is not, therefore, *caused* by the drain of lactation, as has been supposed. Dr. Ashwell, in considering the complaints developed by undue lactation, makes no mention of any malady similar to the nursing sore mouth affection. We thus establish one point in our investigation, viz: that the disease is not an affection peculiar to *nursing* women.

Again: another fact of much importance is derived from Dr. Miner's statement in the discussion of the subject before the Hartford Medical Society, viz: "that he knew a severe epidemic of it in Berkshire Co., Mass., in 1832, (the year of the cholera,) and another at Middletown, Ct., in 1836." The fact established is, the greater frequency of the disease in certain years, amounting to an epidemic in some localities. This accords precisely with our own observations; and if the constitution of those years, as to temperature, snow, rains, frosts, state of the crops and fruits had been given, as called for by our circular, very important deductions, we opine, might be drawn from the premises. In the absence of said particulars we must depend upon our own knowledge of the matter.

The winter of 1831-2, was the coldest winter, according to our recollection, we have ever experienced. The harbor of Baltimore, where we then resided, was closed by ice about four months—the Chesapeake Bay was almost frozen across at Annapolis, a circumstance which was then stated in the public prints to have occurred but *once* since the settlement of Lord Baltimore's colony—the harbor of New York it was conjectured might be closed by the ice that winter—we participated in a sleigh-ride, the thermometer at zero, in the month of April, 1832, and the snow well nigh a foot deep. The Asiatic cholera swept over the United States in the summer of 1832, and what influence the very rigorous winter and retarded spring exerted upon the human constitution in the United States toward rendering it liable to attack, has never been inquired into. That the constitution of the seasons and state of the crops and fruits have very great influence over epidemics there can be no manner of doubt; and while our observations tally with those of Dr. Miner as to the occasional epidemic prevalence of nursing sore mouth, we can as emphatically declare that its epidemic manifestations occur *invariably* after cold winters and retarded springs, accompanied with a scarcity of vegetable supplies. The coinciding fact, therefore, of nursing sore mouth occurring in epidemic form, in Berkshire Co., Mass., a cold mountainous region, in 1832, after an uncommonly rigorous winter and a cold, retarded spring, is another point made in our inquiry.

As to the epidemic of 1836 at Middletown, Ct., this is not quite so clear. We incline to the opinion that Dr. Miner is mistaken in the date, and that it occurred in 1835, the year of its eqidemic occurrence in the West, complicated with other epidemics, as will more fully appear in our chapter on the topography of Illinois, where we discuss the metoric phenomena and constitution of those epidemic years.

In regard to the treatment given by Dr. Ellsworth, we wish particular note to be taken of the good support by way of food, porter as a drink, (full of carbonic acid,) and the preparations of *soda*, *lime*, *iron*, etc., recommended. There is application yet to be made of the principle before we have done, illustrative of the why and wherefore of the efficacy of these acids, salts, alkalies and tonics, combined with wholesome nutrition.

*Dr. Hall's Reply.—Glasgow, Jan.* 26, 1854.—DEAR SIR: Your circular letter bearing date Dec. 32, has been in my possession several weeks, and would have received an earlier response but for the presence of other engagements. Upon its reception I bestowed upon it a careful perusal, and cannot refrain from expressing a hearty commendation of the enterprise you have embarked in, and, so far as any co-operation upon my part may conduce to the results to which your investigations are directed, it is most cheerfully granted. In responding to your inquiries, I have to mention two circumstances which I very much regret should exist—in the first place, the instances of this anamalous affection have been limited; but more especially do I regret that my observations in reference to these cases have been without that systematic accuracy, in the absence of which, facts in a great degree become valueless as materials for etiological, pathological, or therapeutical generalization.

During the last three years, which embraces the period of my acquaintance with this, as a distinctive type of disease, I have, in some manner, been connected with the treatment of five or six well-marked instances of "puerperal anæmia," besides several cases of minor importance, in reference to which I have been casually consulted. The gravest case which has presented itself to my observation, occurred in the person of a lady who was a resident of Logan Co., in the southern part of this State, and who was at the time I saw her (Aug., 1853,) on a visit to her friends in this neighborhood. This was a very charactsristic case. The subject, æt. about thirty-eight years, is habitually anæmic, strikingly deficient in the nutritive function, so much so as to present a very pallid, exhausted appearance, and the buccal affection has regularly recurred, in the early period of lactation, since the birth of her second child—having had six, I believe. The infant then at the breast was about four months old, and from the history of the case elicited from the attending physician and her friends, her general debility increased, and her health continuously declined to this period, when the symptoms had become extremely aggravated. She had been confined to her bed and utterly helpless for fourteen or fifteen days when I fiirst saw her—entire buccal membrane covered with aphthous inflammation, with numerous patches of small ulcers, several large ulcers occupying the edges and inferior surface of the tongue, and some isolated spots of ulceration on the inner surface of the lips, profuse salivation, (not mercurial,) much complaint of vitiated taste with anorexia, pulse 125, and very feeble, with a very low grade of febrile reaction of a regularly remitting type, muscular and nervous exhaustion complete, with extreme feebleness of circulation. So distinctly remitting was the accompanying fever, in this case, that it seemed so urgently to demand an anti-periodic, that such a measure was resorted to and met the indication very happily.

This imperfectly-descriptive history is equally applicable to two others, the most malignant cases of the disease, with tne one described, of which I have any knowledge; and in both these instances the subjects were likewise non-residents of this (Barren) county. One, a young married lady of Gallatin, in Tennessee, came near sinking under a protracted attack, the onset of which dated with the establishment of lactation after the birth of her second child. The other subject of this vitiating infirmity is a resident of Louisville, and as I have been informed by her sister, who resides in this place, like the first named case, it has become so much a constitutional vice as to be habitual with each returning period of lactation. The two females last adverted to seem in a good degree to regain and retain their health and vigor throughout the menstrual cycle, but the health of the first is hopelessly dilapidated, though she is measurably free from the essential symptoms of "nursing sore mouth."

The remaining several instances of the affection, which have been presented to my notice, put on a milder form, yet sufficiently serious to become objects of regular medical attention; presenting in a marked manner the conditions of an impoverished circulation, a depraved state of the nutritive functions, with the more specific local lesions stamped with a less or greater degree of distinctness, in accordance with the mildness or intensity of the attack.

As to therapeutical relations, I conceive that these are, to some extent at least, deducible from the manifest pathological features of the disease. I can but regard the diseased condition as constitutional, consisting mainly in a lesion of nutrition. It may become a question as to where the first link in the chain of morbid actions is to be riveted; but as for my own part, I have learned to regard a vitiated action of the organs of primary digestion as a primitive feature in this pathological state, and other symptoms and conditions as more secondary. The circulating medium certainly becomes greatly depreciated in normal elements, and to replace these constitutes a leading indication of treatment. How to accomplish this, or the principles upon which it is to be done, involves details and considerations not consistent with my present purpose to discuss. So far as concerns the lesions of the mucous lining of the mouth, I can regard this only or mainly as a local manifestation of a more general diseased disposition, and this consideration would lead us to attach but a secondary value to topical measures of treatment; this, my experience fully verifies. I have derived manifest advantage from astringent washes, and even caustic solutions applied to the diseased membrane; but they are to be relied on as less important auxiliaries of a judiciously devised constitutional plan. One drachm of sulphite of soda to the ounce of water forms a valuable wash. The blood dyscrasia, which constitutes the peculiar diathesis of this affection, must be corrected by a set of measures addressed to the nutritive and assimilative functions a properly regulated but nutritious diet, exercise adapted to the health of the patient, and attention to the means of promoting and maintaining the healthful functions of the skin. I have found the most satisfactory results to attend the persevering administration of quinine, chalybeates, the mineral acids, and cod-liver oil, with attention to the state of the secretions.

To speak with more especial reference as to the etiology of puerperal anæmia, I am inclined to discard the influence of climate and locality in the causation of the disease, further than the agency they may exercise in lowering the tone of nutritive life. Thus they may become predisponent agencies; but unless there be an inherent defect of constitution, I should be disinclined to attach much consideration to their influence alone. One of the strongest predisposing causes is, a naturally delicate and enfeebled constitution, and whatever depressing influences may operate to foster and still further deprave this natural disposition to the establishment of the anæemic diathesis; nothing further is wanting to give to the disease its distinctive and characteristic development, but the withdrawal, from the already scanty maternal supplies, of such nutritive elements as answer the demands of gestation and lactation.—I have observed in a majority of cases falling under my notice, that the child is very prone to muguet during the existence of the other disease in the mother. How far may the vitiated meterials of nutrition derived from the mother contribute to the development of muguet in the child?

I will now bring to a close this communication, already extended greatly beyond the contemplated limits when I sat down to write. It will afford me decided satisfaction, at all times, to reciprocate favors of this character, and as a beginning I would gladly have pointed out to me a more successful plan of treating malignant epidemic scarlatina, than experience and reading have, as yet, enabled me to arrive at. I am, most respectfully, yours, etc.,

J. P. HALL.

*Remarks.*—The points in Dr. Hall's paper are, the kind of constitution most liable to this affection, viz: feeble, delicate, breeding and suckling women—its constitutional rather than local seat—its pathology in his judgment being a lesion of nutrition—its sporadic appearance in Kentucky and Tennessee—its very marked, grave, and chronic character—its low grade of remittent febrile exacerbations—the ulceration of the mouth and tongue, attended with profuse salivation not mercurial—the impoverished state of the blood the cause of the disease, and to supply the circulation with normal elements the chief indication in the treatment—and the observed fact that the infants at the breast, in the majority of cases, are also affected with sore mouth. In fine, this contribution comes to us with a freshness from the bed-side of observation in this disease that is exceedingly forcible and instructive, and much to our aid and assistance in these researches. When we come to sum up and offer our views of the nature of this affection, having first set forth all the testimony we can find on the subject, the attention of the careful reader will revert to these practical views of Dr. Hall.

*Dr. Judkins' Reply.—Cincinnati, 4th Mo.*, 1854.—DEAR DOCTOR: Thy circular of inquiry relative to "nursing sore mouth," which thou wert so kind as to send me some weeks ago, I have taken the first favorable opportunity to answer.

In the early part of my practice, I do not now remember to have met with this affection. I often met with diseases, over forty years ago, affecting the mucous tissue resembling stomatitis; also an aphthous condition in children in the same membrane.

Within the last twenty years, but more especially within the last ten years, my attention has been more particularly drawn to notice this adult female disease, for I do not remember ever seeing the disease in any other persons than adult women, and in these only while in the state of lactation.

Females of a lax fiber, thin in flesh, rather of anæmic appearance, are those generally whom I have been called upon to treat for nursing sore mouth. I have known some ladies so predisposed to the affection as never to go through a lactation without it, and others with slight hygienic directions, to escape during the second and third lactations, and perhaps to the end of child-bearing; showing that a strong tendency or predisposition exists in some females to morbid derangements and ulcerations of the mucous linings of the primæ viæ. I say hygienic directions, by which I wish to be understood well-aired rooms for lying-in women; pretty good diet after lactation is established; bathing the skin often over the region of the uterus, both anteriorly and posteriorly, with tepid water, and after which staying the muscles of the abdomen with a bandage; patients to be taken out after their infants are three weeks old, to ride in the fresh air when the weather is suitable; and to be allowed to receive the visits of affectionate friends at suitable times, etc.

In tracing the symptoms as they are developed in this disease, it has manifested itself as *sui generis*, and is confined in its locality, incipiently, to the mucous membrane of the primæ viæ. I am led to this conclusion from the symptoms only in the living subject, having never yet made a post mortem examination to prove this position. There appear to be three stages in the regular phenomena of nursing sore mouth, viz: irritation, inflammation, and ulceration. In addition to these there is (as in most idiopathic diseases) a forming or fixing condition in order to bring about the disease itself, and this is indicated by lassitude, debility, and coolness over the body. Shortly after the lady complains of heat and irritation in the mouth, with a preternatural secretion of saliva; then follow red spots on the sides of the tongue and mouth, which, in a few hours, sometimes terminate in ulcers of from half the size of a three-cent piece to that of a fifty-cent piece. Soon after things have developed themselves thus far, the lady complains of pain and tormina all through the bowels, indicating the same altered structure and ulceration in the primæ viæ throughout.

In the second stage fever is observable, and the irritation occasioned by the ulcerations through the track of the first passages keeps it up for some days, say two or three, unless mitigated by some remedy, yet in common incipient cases lactation is but little retarded; but if the disease is permitted to continue a few weeks, general debility and loss of flesh follow, and the secretions become morbid and the milk fails. Generally speaking, from the time that the first irritation is observable below the pylorus, the bowels begin to act preternaturally, and the dejections are commonly of a thin, watery consistence, inclined to light color. I say generally, but there are some exceptions. I have seen cases where the bowels have been confined during the progress of treatment, except when moved by the administration of laxative medicines, but never without pain.

After the disease has been of two or three weeks standing, by examining a recent alvine evacuation, we discover *floating floculi* in the chamber-vessel of a mucous appearance; this, taken in connection with some other of the symptoms, viz: ulceration, heat, burning pain, etc., in such portions of the mucous tissue as can be seen, lead to the belief that there are ulcerations in the mucous linings of the bowels, nearly, if not entirely throughout the whole track, from which the mucous secretions became separated and found their way to sight.

If this state of morbid derangement continues for two or three months, the body becomes emaciated, hectic fever ensues, and, where there is a strumous diathesis existing, we will have tuberculosis developed, with cough, hemoptysis perhaps, and other fatal symptoms to close the scene.

I will now touch upon the important point in the treatment.—Every intelligent physician, with whom I am acquainted, has his favorite prescriptions in this female affection; and as I have been called to prescribe for quite a number of patients, I will only notice what course I have adopted, hoping that when my experience is added to that of others, something may be sifted out that will be of service. There are two prominent indications to be fulfilled in the treatment: in the first place, we must endeavor to correct the ulcerative process or heal the mucous tissues, and in the second, to restore the morbid secretions and disordered functions to a normal standard.

Unfortunately, the cases that have come under my care have mostly been chronic, and hectic symptoms more or less existed, with diarrhœa, tormina, general emaciation, restless nights, suppressed lactation, etc., etc. I begin the treatment by giving bicarbonate of soda, in fifteen grain doses, dissolved in a tumbler of water, three times a day, which soon corrects the *acid* and *acrid* secretions in the first passages; and, in order to avert diarrhœa, I combine about five drops of the tincture of opium with each dose; order the patient to be well bathed in tepid water, once in the twenty-four hours, when the exacerbations of febrile action are at the highest point; regulate the diet, and avoid such articles of fruits and vegetables as have a tendency to irritate the tender granulations with which they may come in contact, or may exert an unhealthy influence over assimilation, but at the same time enjoin a generous, or *good rich diet.* After a few days thus treated, I give the patient one of the following pills three times a day.

℞

| | |
|---|---|
| Nitrate of silver, . . | gr. x. |
| Denarcotized opium, . | gr. iv. |
| Gum camphor, . . | gr. v. |
| Disulphate of quinine, | ʒj. |

M. f. pil. No. xxv.

As the symptoms vary and improve, I suspend in part or altogether the medicinal treatment, as would occur to any physician, watching the effect of remedies, and the turn and change of symptoms of the patient. Under this plan of treatment I have, but in one case for several years, been under the necessity of taking the child from the breast of its mother, though I am aware of the great assistance afforded thereby in the cure. Very respectfully, thy friend, WILLIAM JUDKINS.

[*New York Journal of Medicine.*]

